# Tree edit distance for leaf-labelled trees on free leafset and its comparison with frequent subsplit dissimilarity and popular distance measures

**DOI:** 10.1186/1471-2105-12-204

**Published:** 2011-05-25

**Authors:** Jakub Koperwas, Krzysztof Walczak

**Affiliations:** 1 Institute of Computer Science, Warsaw University of Technology, Nowowiejska 15/19, 00-665 Warsaw, Poland

## Abstract

**Background:**

This paper is devoted to distance measures for leaf-labelled trees on free leafset. A leaf-labelled tree is a data structure which is a special type of a tree where only leaves (terminal) nodes are labelled. This data structure is used in bioinformatics for modelling of evolution history of genes and species and also in linguistics for modelling of languages evolution history. Many domain specific problems occur and need to be solved with help of tree postprocessing techniques such as distance measures.

**Results:**

Here we introduce the tree edit distance designed for leaf labelled trees on free leafset, which occurs to be a metric. It is presented together with tree edit consensus tree notion. We provide statistical evaluation of provided measure with respect to R-F, MAST and frequent subsplit based dissimilarity measures as the reference measures.

**Conclusions:**

The tree edit distance was proven to be a metric and has the advantage of using different costs for contraction and pruning, therefore their properties can be tuned depending on the needs of the user. Two of the presented methods carry the most interesting properties. E(3,1) is very discriminative (having a wide range of values) and has a very regular distance distribution which is similar to a normal distribution in its shape and is good both for similar and non-similar trees. NFC(2,1) on the other hand is proportional or nearly proportional to the number of mutation operations used, irrespective of their type.

## Background

This paper is devoted to distance measures for leaf-labelled trees on free leafset. A leaf-labelled tree is a data structure which is a special type of a tree where only leaves (terminal) nodes are labelled. This data structure is used in bioinformatics for modelling of evolution history of genes and species and also in linguistics for modelling of languages evolution history. Many domain specific problems occur and need to be solved with help of tree postprocessing techniques such as distance measures, consensus trees, clustering. Distance measures play the most important role as they are very often the start point for more complicated techniques. One of such problem is a problem of competing evolutionary hypothesis. In the process of phylogenetic tree reconstruction, different candidate trees may be obtained, the researches have to determine the true tree of life.

Many existing techniques are designed for trees built of the same leafset which is very limiting. Here we focus on techniques that do not require trees to contain the same set of leaves. Previously we introduced the simple z-restriction approach [1] and more sophisticated frequent subsplit approach [2,3]. Here we introduce the tree edit distance designed for leaf labelled trees on free leafset, which occurs to be a metric. It is presented together with tree edit consensus tree notion and some new results for frequent subsplit based dissimilarity measures approach. For the purpose of experimental testing we follow and extend methodology presented in [4]. We use the popular Robinson-Foulds [5] and MAST [6] based distances as the reference measures. The experiments yield very promising results.

## Methods

### Basic Notions

Here we provide the basic notions and the description of some basic operation on leaf labelled trees which were chosen as the basic operation for new tree edit distance measure. Some derived notions are also presented here.

**Leaf-labelled tree **is a tree with labels assigned to its leaves. Unrooted leaf-labelled trees are very often represented as a set of splits [7].

**Definition **[Split] The Split (or Bipartition) *A*|*B *(of a tree *T *with leafset *L*), corresponding to an edge *e *is a pair of leafsets *A *and *B*, which originated from splitting tree *T *into two disconnected trees, whilst removing an edge *e *from a tree *T*, *A *∪ *B *= *L*. If |*A*| = 1 or |*B*| = 1, the split is trivial.

In this paper, we will refer to the leafset of a given split *s *as *L*(*s*). The set of splits corresponding to each edge builds a unique representation of a given tree. We will refer to the set of splits for a given tree as *S*(*T*). We will use *s *∈ *S*(*T*), or *s *∈ *T *to denote that split *s *occurs in tree T.

**Definition **[Contraction]

The Contraction of a tree T is obtained by removing a chosen internal edge from tree T and identifying adjacent nodes of the contracted edge.

**Figure 1:**
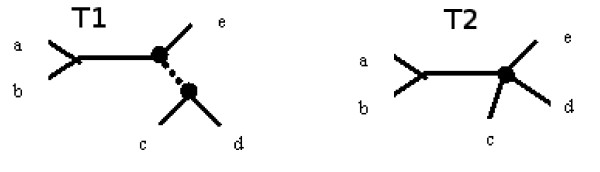
**Contraction operation**.

Because split corresponds to edge (provided that no internal edges of degree two occur), so a contraction may be realised by removing a split from a splitset that represents the given tree. Figure 1 illustrates a contraction operation, and the splitsets are as follows:

*T *1 : *a*|*bcde*, *b*|*acde*, *c*|*abde*, *d*|*abce*, *e*|*abcd*, *ab*|*cde*, *abe*|*cd*

*T *2 : *a*|*bcde*, *b*|*acde*, *c*|*abde*, *d*|*abce*, *e*|*abcd*, *ab*|*cde*

T1 is called a refinement of T2, however T2 is also a subtree of T1 (in more general terms), therefore we will say that T2 is c-subtree of T1.

**Definition **[c-cubtree] A C-subtree of tree T is a subtree where only a contraction operation has been used to construct the c-subtree from its supertree T.

**Definition **[Pruning] Pruning is the operation of removing a chosen leaf from a tree, and afterwards removing the nodes of degree two (which is called forced contraction). The pruning operation can be illustrated on a set of splits as the process of removing leaves from splits, and then removing duplicate splits and not-valid splits, which corresponds to forced contraction.

**Figure 2:**
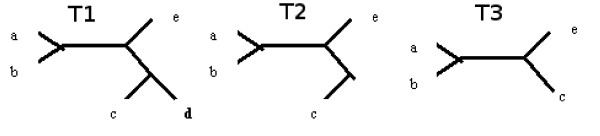
**Pruning operation**. T1 - input tree, T2 - tree where leaf d was removed, T3 - tree after additional forced contraction.

Figure 2 illustrates a pruning operation, where T2 is the process of removing leaf d (a node of degree two emerges after this) and finally T3 is a tree where a forced contraction has also been applied.

*T*1 : *a*|*bcde, b*|*acde*, *c*|*abde*, *d*|*abce*, *e*|*abcd*, *ab*|*cde*, *abe*|*cd*

*T*2 : *a*|*bce*, *b*|*ace*, *c*|*abe*,- |*abce*, *e*|*abc*, *ab*|*ce*, *abe*|*c*

*T*3 : *a*|*bce*, *b*|*ace*, *c*|*abe*, *e*|*abc*, *ab*|*ce*

T3 is called an induced subtree of T1, however here we will call it a p-subtree.

**Definition **[p-subtree] A P-subtree PS of a tree T is a subtree where only a pruning operation is allowed to construct the subtree PS from its supertree T.

**Definition **[restricted tree, z-restricted tree, induced subtree] A z-restricted tree *T^z ^*(alternatively denoted as *T*|*z *in the literature and also called an induced subtree) of a tree T on leafset z, is a p-subtree of T where all leaves not in *z *were pruned. In this paper we use both *T^z ^*and *T*|*z *notations, the second one is more popular and clearer, however it sometimes conflicts with the split notation.

**Definition **[Restricted Split Equality(z-equality)] [1] Splits *s*_1 _and *s*_2 _are restrictedly equal on leafset *z *if their z-restricted versions on leafset *z *are equal.(1)

**Figure 3:**
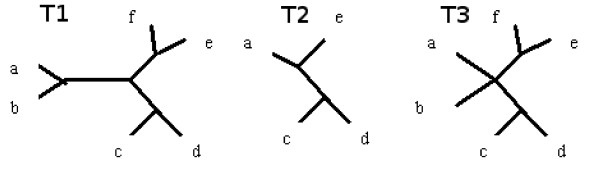
**p-subtree and c-subtree**. Tree together with it's p-subtree(restricted subtree on z = acde) and c-subtree(edge *ab*|*cde f *was removed).

Figure 3 illustrates a more complicated tree together with its p-subtree(restricted subtree on z = acde) and c-subtree.

In [2] we have introduced the subsplit term which is used for the distance and consensus methods discussed later in this paper.

**Definition **[Subsplit and supersplit] [2] Split *s*_1 _is a subsplit of *s*_2 _and *s*_2 _is a supersplit of *s*_1 _iff *s*_1 _is restrictedly equal to *s*_2 _on the leafset of *s*_1_, and the leafset of *s*_1 _is a subset of the leafset of *s*_2_.(2)

This can be presented alternatively as:(3)

#### Common information extraction techniques

**Figure 4:**
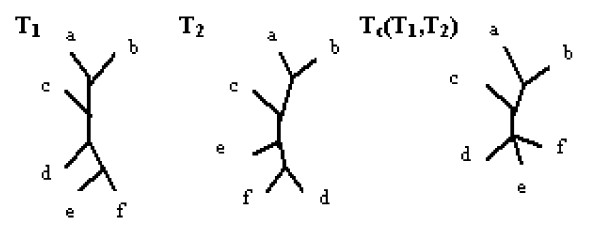
**Strict consensus tree**. Two leaf-labelled trees together with their strict consensus tree.

**Definition **[The strict consensus tree] [8] The strict consensus tree is defined in terms of splits. The strict consensus tree is a tree constructed of all splits common to all trees in a given pro le of trees. Figure 4 presents two trees together with their strict consensus tree.

Splits from T1: *a*|*bcdef*, *b*|*acdef*, *c*|*abdef*, *d*|*abcef*, *e*|*abcdf*, *f*|*abcde*, *ab*|*cdef*, *abc*|*def*, *abcd*|*ef*

Splits from T2: *a*|*bcdef*, *b*|*acdef*, *c*|*abdef*, *d*|*abcef*, *e*|*abcdf*, *f*|*abcde*, *ab*|*cdef*, *abc*|*def*, *abce*|*fd*

The common splits of these trees, which build the strict consensus tree, are as follows: *a*|*bcdef*, *b*|*acdef*, *c*|*abdef*, *d*|*abcef*, *e*|*abcdf*, *f*|*abcde*, *ab*|*cdef*, *abc*|*def*.

Because the concept of a consensus tree is very strict, for many trees, a consensus tree can easily become a star (a tree built of only trivial splits). In order to deal with this problem, many variations of consensus trees have been proposed, among others, a majority rule consensus tree.

**Definition **[Majority rule consensus tree] The majority rule consensus tree is built from splits that occur in the majority of trees.

**Definition **[Maximum Agreement Subtree (MAST)] [6] For a given pro le of leaf-labelled trees *T*_1_,.... *T_n_*, the Agreement Subtree is a tree for which *T_A _*= *T*_1_|*x*··· = *T_n_*|*x *for given x, where *x *⊆ *L*(*T*). The Maximum Agreement Subtree is an agreement subtree with a maximum number of leaves [6].

**Figure 5:**
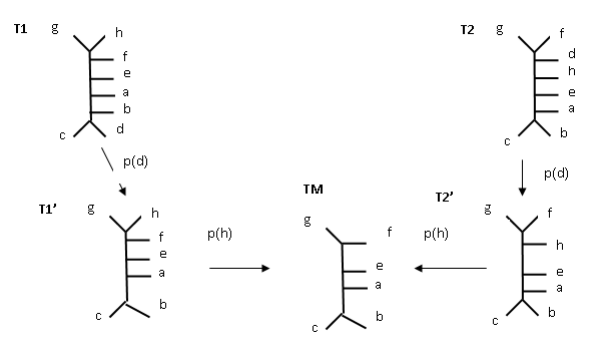
**MAST**. Two leaf-labelled trees together with their MAST on a, b, c, e, f, g.

An example of a MAST can be seen in Figure 5. In Figure 5, T1 and T2 are the input trees, the leaf d is removed from both trees resulting in T1' and T2' respectively. Finally the leaf *h *needs to be removed to achieve an identical tree TM, which is a maximum agreement subtree of T1 and T2.

Several versions of the MAST problem exist like RMAST, which considers only rooted trees, or UMAST for general unrooted trees.

A MAST problem without any restrictions is generally NP-hard [7]. However when the degree of one input trees is limited, then the algorithm is polynomial [7]. Also, when the number of trees is limited to two, then the algorithm is also polynomial.

#### Distance measures

**Robinson - Fould distance **[5] originated from phylogenetic analysis. It is defined as the difference between the number of all splits and the number of splits shared by compared trees. As it was proposed for phylogenetic trees, it is defined for leaf-labelled trees with the same leafset. The R-F distance between two trees *T*_1 _and *T*_2 _with a set of splits *S*_1 _and *S*_2_, respectively, is as follows:(4)

For example, in Figure 4:

*d_R - F _*(*T*_1_, *T*_2_) = 2

**The tree edit distance **[9], [10] between T1 and T2 is defined as the minimal cost of editing operations needed to insert a node, delete a node and relabel a node that transforms T1 to T2. It is based on the concept of edit distance for strings. Tree edit distance was defined for node-labelled and edge-labelled trees. The distance has nice features, it is intuitive, it does not require that compared trees have the same set of leaves. However for trees with leaves which are only labelled, it cannot be used directly. Some artificial internal node labelling is required to use it for such trees, which makes it less intuitive. This distance has not been popular for leaf-labelled/phylogenetic trees. However, in our opinion the idea of edit distance, can be applied to leaf-labelled trees, provided that the editing operations that are selected are natural for them. Such an approach can lead to better distance measures for leaf-labelled trees than existing measures. Such an approach will be presented later in this paper.

**The MAST distance **between trees T1 and T2 is the number of leaves that need to be removed to obtain the Maximum Agreement Subtree.

For the trees from Figure 5, *d_mast _*= 2.

### Representative Splitset and derived similarity measure

Here, we recall the basis of our **representative splitset **approach, which is the foundation for a new consensus technique and new similarity measure, applicable to trees where the leafset may vary without discarding any information. For the detailed information see [2].

#### Notion of Representative Splitset

**Definition **[Frequent subsplit] Frequent subsplit s with support minsup in a profile of trees is a split that is a subsplit of at least one split in at least minsup of trees. The minsup parameter is called the minimal support. It may be an absolute value which denotes the minimum number of trees in which the split is supposed to be found (as a subsplit). It can also be given as a relative value, where it is a minimal percentage of the trees in which the split is supposed to be found.

**Figure 6:**
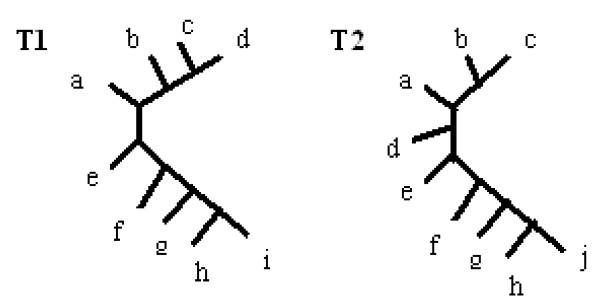
**Sample trees on a different leafset**. Two leaf-labelled trees on a different leafset.

Consider the trees shown in Figure 6, which are represented as follows:

*T*1 : *cd*|*abefghi*, *bcd*|*aefghi*, *abcd*|*efghi*, *hi*|*abcdefg*, *ghi*|*abcdef*, *fghi*|*abcde *plus trivial splits

*T*2 : *bc*|*adefghj*, *abc*|*defghj*, *abcd*|*efghj*, *hj*|*abcdefg*, *ghj*|*abcdef*, *fghj*|*abcde *plus trivial splits

According to our approach, we count the number of trees in which the split occurs (as a subsplit of any split), rather than counting the number of splits, of which it is a subsplit. For example, in Figure 6: *abcd*|*efgh *has the support 2/2 (100%), because it occurs in both trees: in the first one as a subsplit of *abcd*|*efghi*, and in the second one as a subsplit of *abcd*|*efghj*. The argument for counting trees rather than splits is that there may be some subsplits that occur frequently as subsplits of many splits, but only in one tree. Such trees are considered uninteresting.

**Definition **[Representative splitset] Representative splitset - a set that contains maximal frequent subsplits *s*, i.e. such that there is no other frequent subsplit *s_x _*that is also a supersplit of *s*.

**Definition **[strict representative splitset SFS] The strict representative splitset *SFS *is a representative splitset with minsup = 100%. More formally, *SFS *can be represented as follows:(5)

where(6)

**Definition **[Majority-rule representative splitset MRFS] The Majority-rule representative splitset is a representative splitset with minsup = 50%.

#### Frequent Splitset Interpretation

It is clear that, from the splits of FS, we cannot directly construct one tree because the splits in general have different leafsets.

The full reasoning about frequent interpretation was provided in [2]. Here we just recall the conclusions which were derived from the split compatibility Definition and use the fact that from a compatible set of splits a tree can be built:

**Conclusion **1: For each distinct leafset *z *from frequent splitset (FS) with a support greater than 50%, a tree can be built. The tree is built on z-restricted versions of those splits from FS having a leafset as a superset of *z*. Therefore the frequent splitset (minsup > 50%) can be represented as a set of trees. In particular, it affects the strict and majority-rule frequent splitset.

**Conclusion **2: Each split from the frequent splitset discussed above will occur in at least one tree, in a restricted form.

**Conclusion **3: Conclusions 1 and 2 are also true for a tree based on the intersection of all the distinct leafsets from the frequent split-set.

**Conclusion **4: The set of trees resulting from the frequent splitset will also contain a consensus tree, provided that the input dataset of trees was built on the same leafset.

**Figure 7:**
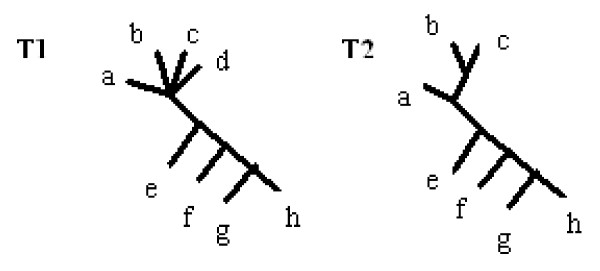
**Illustration of strict frequent splitset**. Two trees built from strict frequent splitset of trees from Fig. 6.

For example, as the strict-frequent splitset of trees from Figure 6 contains splits built on two distinct leafsets: *abcdefg *and *abcefg *the intersection of these leafsets is equal to the second leafset. Therefore, this strict-frequent splitset will be illustrated by two trees as shown in Figure 7.

Strict-frequent-set: *abcd*|*efgh*, *gh*|*abcdef*, *fgh*|*abcde*, *bc*|*aefgh*, *h*|*abcdefg a*|*bcdefgh*, *b*|*acdefgh*, *c*|*abdefgh*, *d*|*abcefgh*, *e*|*abcdfgh*, *f*|*abcdegh*, *g*|*abcedfh*

**Figure 8:**
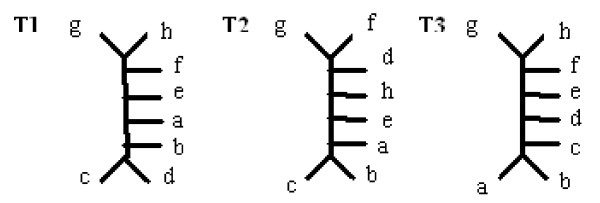
**Sample trees on the same leafset**. Three leaf-labelled trees on the same leafset.

**Figure 9:**
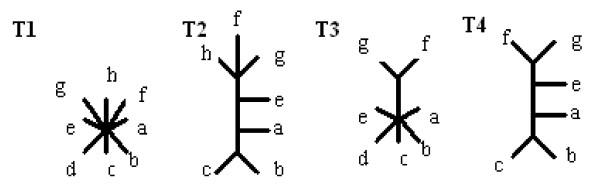
**Illustration of strict frequent splitset**. Trees built from strict frequent splitset of trees T1 and T2 from Fig. 8.

For a more difficult example, let us look at trees *T*_1 _and *T*_2 _from Figure 8: Here, we have three distinct leafsets: {*abcde f gh*} {*abce f gh*} {*abcde f g*} and the intersection: {*abce f g*}. Therefore as a visualisation we present four trees on these leafsets, as shown in Figure 9.

#### FS-based Dissimilarity Measure

Basing on frequent subsplit notion we defined a dissimilarity measure between two trees (or splitsets) [3]. It is not only applicable to trees with different leafsets but also gives more intuitive results for trees with the same leafset:(7)

where *SFS *is a strict-frequent splitset and is the modified sum of both splitsets, which means that, if for splits *s*_1 _∈ *S*_1_, *s*_2 _∈ *S*_2 _, *s*_1 _is a supersplit of *s*_2_, only the supersplit (*s*_1_) is included in the result. Formally, it can be represented as follows:(8)

**Figure 10:**
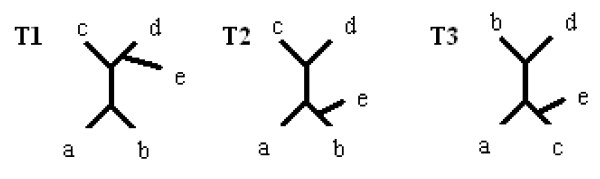
**Sample trees on the same leafset**. Three different trees on the same leafset.

Such a measure determines the dissimilarity on the basis of how many subsplits they share in common. Let us compare this measure to the most popular: R-F distance. Consider the example from Figure 10:

*SFS*(*T*_1_, *T*_2_) = *trivial*(5) + *ab*|*cd*, *SFS*(*T*_1_, *T*_3_) = *trivial*, *SFS*(*T*_2_, *T*_3_) = *trivial*

*dRF *(*T*_1_, *T*_2_) = 4, *d*(*T*_1_, *T*_2_) = 1 - 6/9 = 4/9

*dRF *(*T*_2_, *T*_3_) = 4, *d*(*T*_2_, *T*_3_) = 1 -5/9 = 5/9

It is clear that the R-F distance states that *T*_1 _and *T*_2 _are both as dissimilar as *T*_2 _and *T*_3 _whilst our measure arrives at a different result, which is an intuitive result since both *T*_1 _and *T*_2 _share a common non-trivial subsplit *ab*|*cd*. For trees on a different leafset, the R-F distance does not work at all whilst our measure does.

The main drawback of this measure is that it is not a metric, however it achieves very good statistical characteristics and clustering results as described in the Results section. In this paper the method was compared to R-F, MAST and edit distance in the series of experiments.

### Tree Edit Distance and Tree Edit Consensus for Leaf-Labelled Trees

#### Tree Edit Distance for Leaf-Labelled Trees

**Figure 11:**
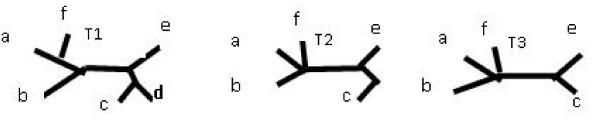
**Sample edit script**. Example of transforming tree T1 into T3 using edit operations.

In the following sections we define a new distance and consensus notion based on editing operations on leaf-labelled trees. We choose contraction and pruning as editing operations for leaf-labelled trees. If tree T3 is a subtree of T1, where both pruning and contraction operations are allowed, then we call it a pc-subtree or edit subtree. An example of transforming tree T1 into T3 using editing operations is shown in Fig. 11.

**Definition **[Edit script] An Edit script *S*(*T*1, *T*2) for leaf-labelled trees *T*1 and *T*2 is the pair of subscripts *S*(*T*1) and *S*(*T*2) which are sequences of editing operations including contraction and pruning, which can be applied to the selected input trees T1 and T2 to unify them. *S*(T1, T2) = *S*(*T*1) ∪ *S*(*T*2). The subscripts *S*(*T*1), (*S*(*T*2)) are uni-directed which means that by using S(*T*1), we can modify *T*1 to obtain the tree that is a unification of *T*1 and *T*2, but not necessarily in the opposite direction.

**Definition **[Edit script Cost] The cost of an edit script *Cost*(*S*) is the sum of the defined costs of editing operations: contraction and pruning where *Cost*(*e*) = *cost_c _*if e is a contraction and *Cost*(*e*) = *cost_p _*if e is a pruning operation. Forced contractions may be counted or not, depending on application.

**Definition **[Tree edit distance for leaf-labelled trees]

Having defined positive value costs for contraction and pruning operation, the tree edit distance for leaf-labelled trees T1 and T2 is the minimal cost edit script *d*(*T*1, *T*2) = *minCost*(*S*), where forced contractions are counted as normal contractions. Note that in order to keep the resulting tree as leaf-labelled tree only contractions that correspond to non-trivial split are performed, unless it is a forced contraction - than trivial split may also be contracted to remove split with duplicate representation. In this paper, we focus on the edit distance which counts forced contractions. Thanks to this property, we can prove that it is a true metric.

However, there is also an interesting variant of the edit distance where a forced-contraction is ignored. The metric property of such a variant is yet to be verified, the measure will also be considered in experiments due to its interesting features.

**Definition **[No Forced Contraction Disimilarity Measure for leaf-labelled trees] Having defined positive value costs for contraction and pruning operation, the No Forced Contraction Disimilarity Measure for leaf-labelled trees T1 and T2 is the minimal cost edit script *d*(*T*1, *T *2) = *minCost*(*S*), where forced contractions are ignored.

#### Tree Edit Distance versus R-F Distance and MAST

As mentioned earlier, comparing distance measures is not a trivial task. Here, we provide a subjective opinion about why this measure is better than others, however an objective statistical comparison will be provided in the Results section.

The R-F and MAST distances have some drawbacks which have emerged from the fact that the R-F distance may use only contraction operations and MAST uses only pruning operations and forced contractions. There are of course some cases when all three distances perform equally well, as in the example of Figure 1, where for Trees T1 and T2, the R-F Distance = 1, MAST = 1, Edit Distance = 1.

#### R-F Distance Drawbacks

**Figure 12:**
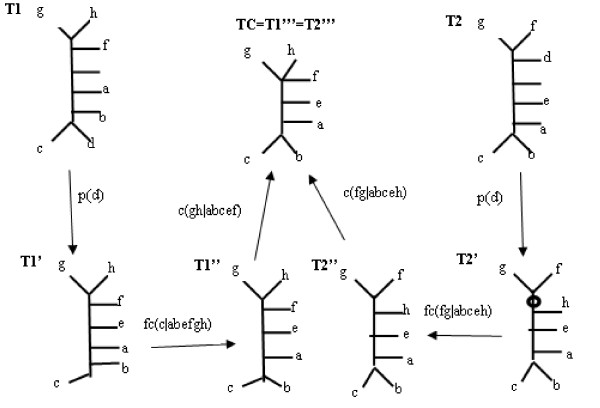
**Edit-consensus tree**. Trees T1 and T2 together with their edit-consensus tree TCe.

1) The first drawback of the R-F distance is that it is totally useless for leaf-labelled trees on a free leafset. For example, Figure 2 shows two trees on a different leafset (T1 and T3). The R-F distance is undefined here. It can be seen that the removal of one leaf and the internal edge is sufficient to make the two trees identical. In this case, both the MAST and Edit Distance can be used as MAST can be extended to support a free leafset, and the Edit Distance is naturally suitable for a free leafset. MAST and Edit distances will provide different values: MAST distance = 1, Edit Distance = 2. These differences will be discussed in the following section.

2) The second drawback of the R-F distance is that even if the trees are on the same leafset, one noisy leaf may cause the trees to be considered totally different (all splits must be removed). Removal of one leaf may significantly reduce the distance between trees. Such a situation is illustrated in Figure 12. Trees T1 and T2 look totally different, in terms of the R-F distance, because of leaf *d*, thus all non-trivial splits must be removed (all the information!) in order to make them identical. R-F Distance = 10, however, removing only leaf *d *would result in trees differing by only 2 splits! Therefore, the MAST distance equals 2 and the Edit Distance equals 6.

#### MAST Distance Drawbacks

**Figure 13:**
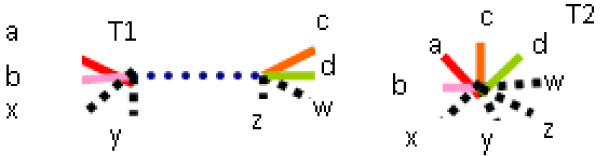
**Two trees that differ by one internal edge only**.

**Figure 14:**
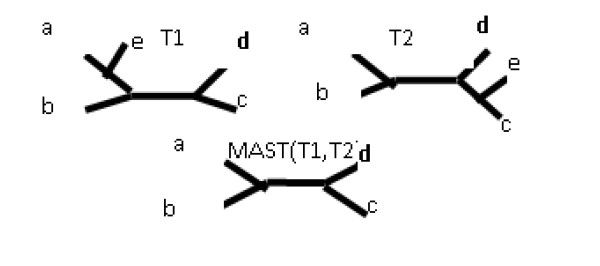
**MAST**. Example of the MAST tree between T1 and T2.

1) The first drawback of the MAST distance occurs when the trees are similar except for one internal edge as in Figure 13. In this case, the MAST distance would equal 3 as it requires the removal of at least 3 leaves in order to make the trees identical. However, both the R-F and Edit distances may just remove one edge, thus the R-F Distance would equal 1 and the Edit Distance would equal 1.

2) The second drawback is that MAST counts only the leaves that are removed from both input trees. If it is allowed to also count leaves that are present in only one tree in order to support a different leafset, then the distance will ignore some subtle changes. For example, in Figure 14, the distance between T1 and T2 is identical as in the example with T1 and MAST(T1, T2), which is obviously incorrect. The solution to this problem would be to count the leaf twice if it is removed from both trees and count it once if is removed from one tree. This solution would not however fix another problem: MAST completely ignores forced contractions. Therefore, some subtle differences may again be missed. For example the MAST distance between T1 in Figure 1 and T2 in Figure 2 is the same as between T2 from Figure 1 and T2 from Figure 2, which is again incorrect. In this case, the MAST distance would be equal to 1, but the Edit Distance equals 2 and 1 respectively.

#### Edit Distance Advantage

In the previous sections, we showed the drawbacks of RF and MAST distances and showed that the Edit Distance is better because:

• it can be used for trees on a free leafset

• it can distinguish differences where the MAST distance cannot as it can use both contraction and pruning.

Let us compare the values of these distances. The Edit Distance is easily compared to the R-F distance, provided the same cost of contraction is used to count both distances. The only difference between them is that the R-F distance cannot use pruning. However, it is impossible to compare the values directly to MAST as this distance is not well defined if the leaf is removed from one tree only, and the cost of forced contractions is ignored. Therefore, in order to compare the values, we will use the R-F distance (denoted here as the c-distance) and instead of MAST, we will count the cost of each pruning operation and forced contraction (denoted here as the p-distance). Some distance values are presented in Table 1.

**Table 1:** Values of c, p and edit distances for various examples.

Fig (trees)	c-dist	p-dist	Edit-dist
1(T1, T2)	1	2	1
2(T1, T3)	-	2	2
11(T1, T3)	-	5	3
14(T1, T2)	4	4	4
13(T1, T2)	1	4	1
15(T1, T2)	8	7	4

**Figure 15:**
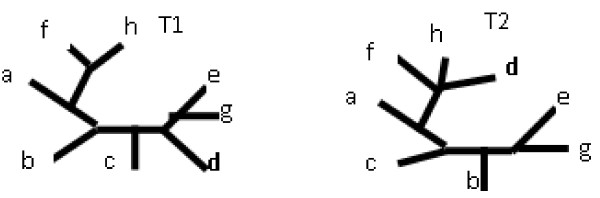
**Two trees on the same leafset where the Edit distance is more suitable than others**.

These results show that, in some situations, pruning operations are better at unifying trees, sometimes contractions are, and sometimes neither performs well. However, there are cases when using both of them is better. To sum up below, there are some cases when one editing operation is better than another: Pruning is better: when the trees are not on the same leafset, then pruning is necessary (figures 2, 11, 14) the trees may be on the same leafset but they contain some noisy leaf (leaf *d *in trees T1 and T2 from Figure 12). Contraction is better: when two significantly large sub-trees are connected directly on one tree, but connected with an additional edge on the second tree (Figure 13). Both operations seem to be equally good when the trees are on the same leafset, there is no noisy data, and the degrees of the nodes are relatively small. Because there are some cases when one operation is better than the other, a distance based on both operations shall be better than one based on only one operation. A distance constructed in this way will choose the most appropriate editing operations. For example, consider Figure 15, the c-distance equals 8 (all nontrivial splits), p-distance (d and b in both trees plus 3 forced contractions) equals 7, pc-distance (i.e. edit distance with unitary costs) equals 4 (d in both trees plus 2 splits)

#### Cost Manipulation

The difference between the Edit Distance and other distances is visible especially when the cost of operations is not the same. Although in some cases both operations can be equally good, one may prefer for example contraction over pruning in some cases. The motivation can be for example the need to have as many leaves as possible in the tree edit consensus. Therefore, our distance uses the costs of editing operations. For example, consider the trees T1 and T2 in Figure 12, and assume that the pruning cost equals 2 and the contraction cost equals 1. For these trees, if only the pruning operation is used, then the p-distance equals 12 (removal of d and h from both trees and forced contractions), if only a contraction is used then the R-F-distance equals 10 (removal of all non-trivial splits in both trees), however if the Edit distance is used then the distance is equal to 8 (removal of d from both trees, then removal of two differencing splits). The edit script is also illustrated, with its semi-products (T1' and T2'). If we assume the following costs: 3 for prunning and 1 for contraction, then the Edit Distance would consist of contraction operations only, and the distance would be equal to 10.

#### Tree Edit Distance Metric Proof

In order to show that our measure is a true metric, the following conditions shall be proved:

• *d*(*T*_1_, *T*_2_) = 0 ⇔ *T*_1 _= *T*_2_

• *d*(*T*_1_, *T*_2_) = *d*(*T*_2_, *T*_1_)

• *d*(*T*_1_, *T*_2_) + *d*(*T*_2_, *T*_3_) ≥ *d*(*T*_1_,*T*_3_)

The first two conditions are met by definition: The minimal edit script that unifies T1 and T1 contains no operations, therefore the distance is equal to 0. On the other hand, if two different trees T1 and T2 may be unified only by applying some editing operations, and because cost must be positive-valued, then the distance for different trees cannot have the value 0.

As the Definition states that the distance is the minimal cost of unifying two trees, by applying the editing operations either to T1 or T2, it is therefore symmetric by Definition.

The third condition is slightly more complicated and requires more explanation:

**Lemma **: Having the edit scripts corresponding to distances *d*(*T*_1_, *T*_2_) and d(*T*_2_, *T*_3_), we can unify trees T1 and T3 using the same operations as on both scripts (or a subset of them).

Proof:

Lets denote:

• TPCX the tree edit consensus (unification) of trees T1 and T2.

• TPCY the tree edit consensus (unification) of trees T2 and T3.

• Sx1(T1) = TPCX - the edit subscript that transforms T1 to TPCX

• Sx2(T2) = TPCX - the edit subscript that transforms T2 to TPCX

• Sy2(T2) = TPCY - the edit subscript that transforms T2 to TPCY

• Sy3(T3) = TPCY - the edit subscript that transforms T3 to TPCY

**Figure 16:**
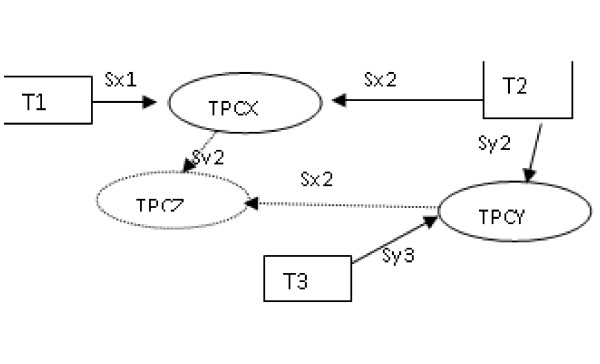
**Illustration for proof of lemma 1**.

The mentioned artefacts are presented in Figure 16.

Because *S*1(*S*2(*T*)) = *S*2(*S*1(*T*)), which will be shown in the next section, we have:

*Sx*2(*Sy*2(*T*2)) = *Sy*2(*Sx*2(*T*2)) and *Sx*2(*TPCY*) = *Sy*2(*TPCX*), because *Sy*2(*T*2) = *TPCY *and *Sx*2(*T*1) = *TPCX*

Therefore, there exists some tree TPCZ, such that *Sx*2(*TPCY*) = *TPCZ *and *Sy*2(*TPCX*) = *TPCZ *(dotted line on Figure 16), which can be obtained from T1 and T3 with at most the same number of operations as unifying T1 with T2 and T2 with T3.

**Theorem: **The tree edit distance for leaf-labelled trees meets the third metric condition.

**Proof: **Due to Lemma, presented earlier, there exists an edit script *S*(*T*1, *T*3) that can unify trees *T*1 and *T*3 at the same cost (or less) than the sum of: *d*(*T*_1_, *T*_2_) + *d*(*T*_2_, *T*_3_)

*Cost*(*S*(*T*1,*T*3)) <= *d*(*T*_1_, *T*_2_) + *d*(*T*_2_, *T*_3_)

and because

*d*(*T*_1_, *T*_3_) <= *Cost*(*S*(*T*1, *T*3))

therefore:

*d*(*T*_1_, *T*_3_) <= d(*T*_1_, *T*_2_) + *d*(*T*_2_, *T*_3_)

#### Edit Subscript Order

In this section we show that for a given edit subscript (i.e. a set of operations on one tree), changing the order of operations in it will not change the resulting tree. Therefore, it will also not increase the costs. In order to show this, we need to show that if edit script consists of operations: *p*_1_,..., *p*_*n *_and *c*_1_,..., *c_m _*then the changing order of operations does not change the result.

Let us assume that a tree is represented with two sets *E*(*e*_1_... *e_n_*) - a set of internal edges referring by non-trivial splits and *L*(*l*_1 _... *l_n_*) - a set of leaves. Let us consider edit script ES that transforms *T*_1 _represented with *E*_1_, *L*_1 _to tree *T*_2 _represented with *E*_2_, *L*_2 _and *E*_2 _⊆ *E*_1 _and *L*_2 _⊆ *L*_1_. Assume for a moment that we will not handle forced contractions. Under such assumption the edit operations: contraction and pruning operate on a different set of items - edges(referred with splits) and leaves respectively. Therefore position of contraction operation, with relation to pruning operation in edit script (and vice versa) does not affect the result. Additionally, from the set theory - the order of items removal from a set is irrelevant - therefore position of contraction operation with relation to other contraction operation in edit script is irrelevant. The same holds for pruning. This leads directly to conclusion that the order of operations in edit script does not affect the final result.

One may notice that pruning also removes some edges, but only trivial ones, which are not considered in edit distance and may be removed at any time. One may also notice that pruning changes the bipartition representation of all non-trivial splits. It is also not a problem, as the total number of edges is not affected. Although we use split representation very often, here the number of edges is important (not the form of their split representations).

As it was presented earlier in this paper, pruning may occasionally introduce forced contraction (see Figure 2). It does not, however, break the assumption that operations work on different sets and are independent of each other.

Let us represent pruning *p *as the pair of operations *p' *that removes leaf only as assumed earlier and *fc *that performs forced contraction. So each time pruning *p *occurs in edit script it is replaced with either *p' *if there is no forced contraction to perform or *p'*, *fc*. Operation *fc *may be treated as regular contraction operation, with the only difference that it is inserted by pruning. It can also be easily shown that if we change the order from *p'*, *fc *to *fc*, *p' *the result remains the same as the pruning operation does not trigger forced contraction if the appropriate edge was removed earlier in edit script.

The last thing that we mention is the edge matching. Forced contraction removes the edge, which is a duplicate of another edge with respect to their split representation. For example *e*_1 _= *ab*|*cde *and *e*_2 _= *abe*|*cd *will be consider duplicates if leaf *e *is pruned. It may be therefore problematic which edge *e*_1 _or *e*_2 _is in fact removed from set of edges. Therefore if the forced contraction is to be done then we shall treat it as the unification of duplicate edges. That means operations *c*(*e*_1_) and *c*(*e*_2_) can be used exchangeably within the edit script, as the edges are unified sooner or later.

#### Algorithm for Counting Edit Distance of Leaf-labelled Trees

The naive algorithm for this problem can be illustrated as follows:(9)(10)

where *d_s _*is the distance for trees on the same leafset, *T*_2 _- *s *denotes tree T2 with removed split *s*, Δ stands for symmetric difference and k indicates the number of forced contractions that needed to be performed. To keep metric property *cost_fc _*is equal to *cost_c_*. In the equation the following part: cost_p _* |*L*(*T*_1_)Δ*L*(*T*_2_)|+k * *cost_fc _*is in fact the cost of unification of leafset for both trees. The algorithm is therefore exponential with respect to the number of leaves and the number of splits. We modify algorithm on the basis of two observations. The first one is that the order of editing operations is irrelevant therefore the algorithm can try prunning operations before it tries contractions. The second one is that the Edit Distance will never remove the splits that occur in both trees (except forced contractions), which can be easily proved, only differing splits (i.e R-F distance) are considered. After modifications algorithm presents as follows (*L*(*T*1 = = *L*(*T*2)).(11)

The algorithm can also be presented with pseudocode as follows:

function edit_distance(T1,T2, cost_c, cost_p) {

D1 = L(T1) - L(T2) ;

D2 = L(T2) - L(T1) ;

T1'=prune(T1,D1) ;

T2'=prune(T2,D2) ;

//after above operations L(T1') = = L(T2')

cost = (|D1|+|D2|)*cost_p+ cost_c * (how_many_fc(T1,T1',|D1|)+how_many_fc(T2,T2',|D2|))

      + edit_distance_the_same_leafset(T1',T2',cost_c,cost_p);

return cost;

}

function edit_distance_the_same_leafset(T1,T2, cost_c, cost_p){

   minCost = R-F-distance(T1,T2) * cost_c;

   L = L(T1); // L(T1) = = L(T2)

   For each leaf in L

      T1'=prune(T1,leaf);

      T2'=prune(T2,leaf);

      cost_all_fc = cost_c * (how_many_fc(T1,T1',1)+ how_many_fc(T2,T2',1));

      costT = edit_distance_the_same_leafset(T1',T2', cost_c, cost_p)

         + 2*cost_p + cost_all_fc;

      if (costT < minCost) minCost=costT;

   end for

return minCost;

}

function how_many_fc(T1,T1',k) { return |S(T1)| - |S(T1')| +k)

// k parameter is used to prevent counting trivial split contration directly

// associated with leaf removal

function prune (T1,L) - prunes all leaves from set L from tree T1 and

performs forced contractions.

This algorithm is now exponential with respect to the number of leaves. It is possible that this can also be improved so that it has the same complexity as MAST for two trees (which is polynomial), but further investigations are required. For the purpose of this paper, we used a dynamic programming algorithm, where partial results are stored in memory and re-used if necessary. It turns out that the algorithm was required to only count a small part of all possible combinations which also gives grounds for optimism that a better algorithm will be found.

Let us look on a few steps of naive algorithm for trees T1 and T2 from Figure 12

T1:*gh*|*abcdef*, *fgh*|*abcde*, *efgh*|*abcd*, *aefgh*|*bcd*, *abefgh*|*cd*

T2:*f g*|*abcdeh*, *dfg*|*abceh*, *dfgh*|*abce*, *defgh*|*abc*, *adefgh*|*bc*

Trees are built on the same leafset so we may directly calculate *d*_*s *_equation. *d*_*R-F *_(*T*1, *T*2) = 10. Let us remove some leaves (we will not show all of them due to clarity of the presentation): Let us remove *a*, we obtain:

T1':*gh*|*bcdef*, *fgh*|*bcde*, *efgh*|*bcd*, (*efgh*|*bcd*), *befgh*|*cd*

T2':*fg*|*bcdeh*, *dfg*|*bceh*, *dfgh*|*bce*, *defgh*|*bc*, (*defgh*|*bc*)

plus the trivial splits

Curly braces denote splits that will be force-contracted.

*d*_*R-F *_(*T*1*'*, *T*2*'*) = 8, cost of pruning: 2, and cost of forced contractions: 2 Total cost in this path: 12, so the result is worse than *d*_*R-F *_(*T*1, *T*2), and the prunning additional leaves will also not improve the result.

So let us remove *d *instead of *a*

T1':*gh*|*abcef*, *f gh*|*abce*, *efgh*|*abc*, *aefgh*|*bc*, (*abefgh*|*c*)

T2':(*fg*|*abceh*), *fg*|*abceh*, *fgh*|*abce*, *efgh*|abc, *aefgh*|*bc*

*d*_*R-F *_(*T*1*', T*2*'*) = 2, cost of pruning: 2, and cost of forced contractions: 2 Total cost in this path: 6, so the result is better than *d*_*R-F *_(*T*1, *T*2) We may continue with pruning of another leaf to see if we can improve the result more, so we remove *g*

T1":(*h*|*abcef*), *fh*|*abce*, *efh*|abc, *aefh*|bc

T2":(*f*|*abceh*), *fh*|*abce*, *efh*|*abc*, *aefh*|*bc*

*d*_*R-F *_(*T*1*"*, *T*2") = 0, cost of pruning: 2, and cost of forced contractions: 2, cost calculated from previous

step: 4, so the total cost is equal to 8 thus we received a worse result. We will not continue with pruning of other leaves as it will not lead to better result.

Therefore the best cost is 6, and the edit script contains two subscripts:

From T1: p(d), *fc*(*c*|*abefgh*), *c*(*gh*|*abcef*)

From T2: p(d), *fc*(*fg*|*abceh*), *c*(*fg*|*abceh*)

#### Tree Edit Consensus Tree

Similarly, we may define a new consensus method on the basis of editing operations called the Tree Edit Consensus Tree. The Tree Edit Consensus Tree is the maximal (with respect to leaves and edges) common subtree of the input trees, obtained by contraction and pruning operations and is defined as follows:

**Definition **[Tree edit consensus tree (PC-Consensus tree) ] Having defined the positive value costs of contraction and pruning operations, the tree edit consensus for leaf-labelled trees *T*1 ... *Tn *is the minimal cost edit script that unifies these trees. For trees T1 and T2 in Figure 12 the tree edit consensus tree is tree *TC *= *T*1*" *= *T*2*" *Similar to MAST, the tree edit consensus tree is not unique. The experimental assessment of this method was done in PhD thesis [11] and will not be recalled here.

#### Tree Edit Consensus Algorithm

Similar to the edit distance, based on the fact that, if a prunning operation is used it must be used on all input trees and the fact that contraction is performed only for splits that do not occur in all input trees. The naive, dynamic programming algorithm which counts the score of the tree edit consenus may be defined as follows:(12)(13)

where , SCT stands for strict consensus tree, TEC is the Tree Edit Consensus k is the number of forced contractions that needed to be performed and *cost_fc _*is equal to *cost_c_*

The tree edit consensus tree may be obtained by recording prunning operations used along the optimum path. Recorded prunnings must be applied to input trees, and afterwards all unmaching edges must be contracted (strict consensus tree).

#### Quality of similarity measures

The quality of similarity measures is not obvious to estimate. The best possible method would be a method based on external criteria i.e. based on expert knowledge. In biological applications, it could be a comparison of the consensus tree with the true phylogenetic tree. The true tree however is something that is not known. We agree with the opinion presented in [4] that similarity measures are especially difficult to score as they are very subjective about what is similar and what is not. Such a subjective approach to score the distance requires that someone arbitrarily selects the best distance matrix. This matrix is to be compared with the distance matrix achieved with a given similarity measure. A more objective method would be to prove that a given measure is best in some particular applications or may be used to solve some particular problems, like for example the identification of the ancestral paralog position in the paralog families mentioned in [12].

The methods that can be applied to measure the quality of distance measures and consensus techniques can be roughly divided into:

• qualitative methods which try to de ne properties that the given consensus method or similarity measure must meet as in [7]

• quantitative methods which try to measure the quality of consensus or similarity methods such as [13]. They are often based on some assumptions due to the lack of verified domain knowledge

• statistical methods which display the statistical properties of the given method to help an expert score the method instead of scoring it automatically, because the quality of a metric may depend on the application.

In an axiomatic approach, the most common requirement for the similarity measure is that it meets metric properties, or at least pseudo-metric ones.

The quantitative approach is not very suitable for distance measures due to lack of objective criteria. Even if we are supplied with biological data which contain groups of trees and may count for example the proportion of inner-group distances to between-group distances, such an approach is not very trustworthy. This is because we see the effect of the distance measure on selected sets, which may be different for different parts of the tree-space. We also ignore some potential properties of distance, for example that the distance metric may be better for some topologies of trees but worse for others and this observation could give hints on where to use it and where not. Simply put, the quality of a metric may depend on the application.

Except for proving the metric properties of some distances, we choose the statistical approach as described in [4] and perform two kinds of experiment:

• Analysis of distance probability distribution

• Analysis of distance dynamics with respect to number of changes in trees.

In the first approach, we count the distance for a large number of randomly generated unrooted trees according to different distributions and examine the distribution of probability. The details of random generation of rooted trees can be found in [14]) - this method can be adaptable for unrooted trees.

*P*(*d*(*T*1,*T*2) = *k*). We try to determine:

• whether the distance distribution has any regularities, follows any known distribution, which proves that the distance does not work in a random fashion

• whether the distance is well enough discriminative (has a large number of values), whether the discrimination property is equally strong for similar and different trees.

The other approach is to mutate the random tree with different mutation operations and see how the distance changes.

More details about the experiments will be provided in the Results section.

## Results and Discussion

In this section, an experimental evaluation of the proposed methods is presented. For the purpose of experiments, we use the randomly generated trees with different distributions and we evaluate the statistical properties of the similarity measures as described previously in this paper. From our propositions, we decided to evaluate the Tree Edit Distance, No Forced Contraction Similarity Measure called NFC here and the FS-based Similarity measure.

For comparison with existing distance measures we have chosen the R-F and MAST distances. R-F is one of most popular and computionally efficient distances, MAST and R-F are in a way foundations of the Edit measures presented in this paper. We decided not to normalise the values of distances because sometimes normalisation is not obvious (as in the case of the Edit distance). Normalisation is not necessary in the first experiment as we study the distribution rather than absolute values. In the second experiment, the lack of normalisation does not prevent us observing dynamics, it only forbids the spotting of the crossing points of distances. The approach of normalising with the maximum observed value, as used in literature, in our opinion distorts the results, because if the real maximum value is not achieved then the graph is distorted. The only modifications are made with the FS dissimilarity measure, i.e. values are scaled and biased in order to be compared with other distances on the same chart.

In this experiment, trees with 8 leaves are presented, however tests were also performed with trees with up to 17 leaves for unconstrained trees and 12 leaves for binary trees, with similar results being obtained.

### Distribution of Distance Probability

For this test, 1000 pairs of trees with 8 leaves were generated and the distribution of probability *P*(*d*(*T*1, *T*2) = *k*) was examined under different tree generation models, as mentioned previously in this paper. As the Edit Distance and its version with no penalty for forced contraction are parametrizable, various pruning and contraction operations costs were used. In the following experiments, the edit distance with cost of contraction equal to x, and cost of pruning equal to y is denoted by E(x, y), the version with no cost for forced contraction is denoted by NFC(x, y).

### Unrooted binary leaf-labelled trees on the same leafset

**Figure 17:**
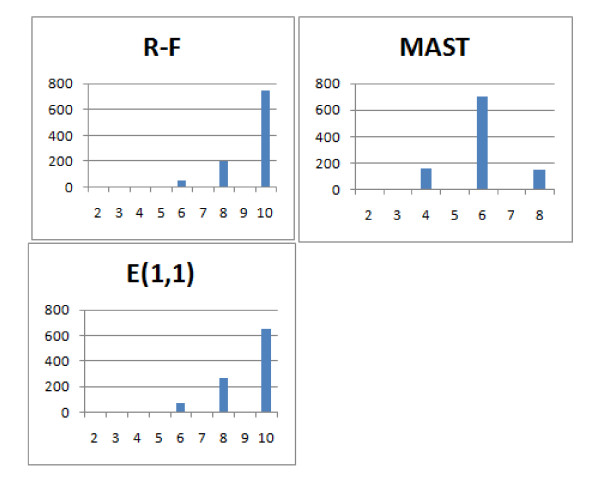
**Comparison of distributions of selected measures**. Comparison of R-F, MAST and E(1,1) distributions.

First consider Figure 17 which shows the R-F, MAST and Edit distances with the cost of pruning and contraction equal to 1. It appears that all of these distances on this dataset took only 4 unique values each. Among them only 3 frequent enough to be visible on the figure. This leads to the conclusion that they are not very discriminative, as the total number of unrooted binary trees with 8 leaves is ≈ 10^4^. The Edit distance and R-F distance behave identically here, the number of occurrences of particular distance value increases asymptotically with the value, which means that these distances are good only for similar trees. On the other hand, MAST is also not very discriminative but it is more reminescent of the normal distribution.

**Figure 18:**
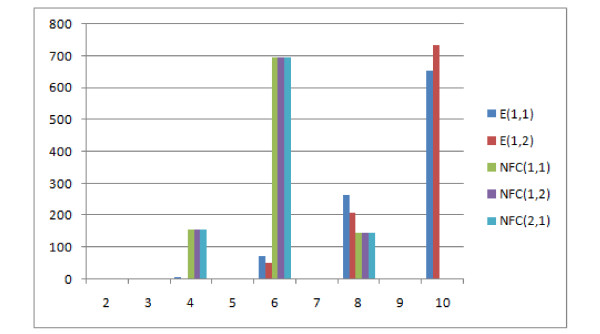
**Comparison of distributions of selected measures**. Comparison E(1,1), E(1,2), NFC(1,1), NFC(1,2), NFC(2,1) distributions, showing the number of obtained pairs of trees (y axis) with certain distance values (x axis) in 1000 trials.

Figure 18 shows that the E(1,1), E(1,2), NFC(1,1), NFC(1,2), NFC(2,1) distributions are similar or identical. Due to the fact that E(1,1) is identical to R-F for these data, they won't be discussed more here.

**Figure 19:**
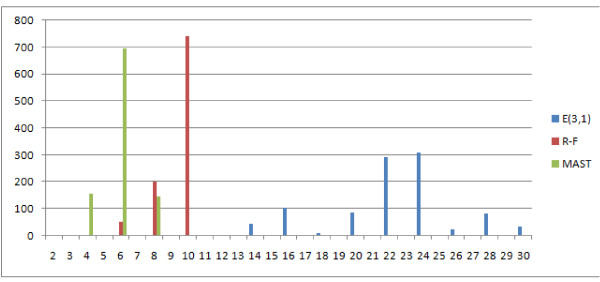
**Comparison of distributions of selected measures**. Comparison E(3,1), MAST and R-F distributions, showing the number of obtained pairs of trees (y axis) with certain distance values (x axis) in 1000 trials.

The distances E(2,1) and E(3,1) are significantly different, especially E(3,1) which is compared to MAST and R-F in Figure 19. The E(3,1) distribution is similar in shape to MAST so it can be used both for similar and distant trees, however it has a wider range of values (11 unique values for E(3,1) as compared to 4 for MAST.)

**Conclusion **The first conclusion is that by modifying the costs of the Edit distance, we can achieve a measure with very well-behaving properties: very discriminative and suitable both for similar and dissimilar trees. Moreover, the similarity of the Edit, R-F and MAST distributions shows that the distance is not accidental.

**Figure 20:**
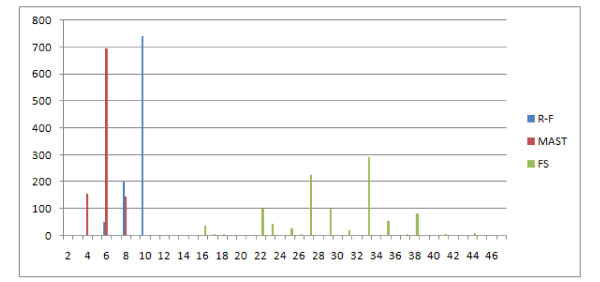
**Comparison of distributions of selected measures**. Comparison of FS dissimilarity measure with R-F distributions, showing the number of obtained pairs of trees (y axis) with certain distance values (x axis) in 1000 trials.

The FS similarity measure is the hardest to interpret (see Figure 20), it also has a wide range of values (19 unique values) so it is discriminative, however the shape of the distribution is very irregular. However, if we merge the low peaks with neighbouring high peaks, we achieve something similar to the R-F distance, i.e increasing with increasing distance value. So the conclusion is that it is discriminative but works better for similar trees than for distant trees. It is worth remembering that this measure is not metric for sure, therefore this may affect its properties.

### Unrooted unconstrained leaf-labelled trees on the same leafset

**Figure 21:**
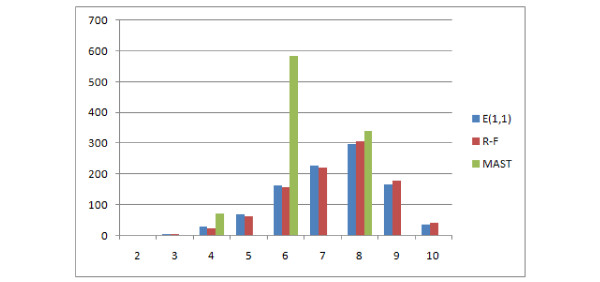
**Comparison of distributions of selected measures**. Comparison of E(1,1) with R-F and MAST distributions, showing the number of obtained pairs of trees (y axis) with certain distance values (x axis) in 1000 trials.

**Figure 22:**
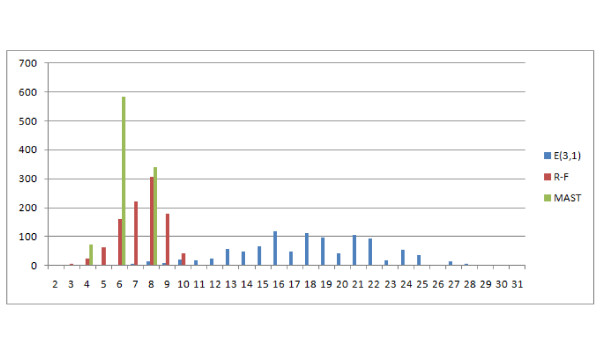
**Comparison of distributions of selected measures**. Comparison of E(3,1) with R-F and MAST distributions, showing the number of obtained pairs of trees (y axis) with certain distance values (x axis) in 1000 trials.

**Figure 23:**
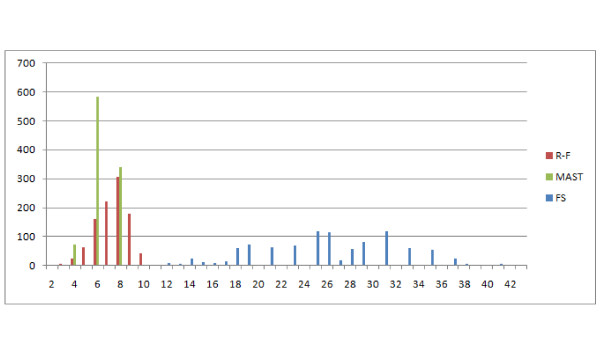
**Comparison of distributions of selected measures**. Comparison of FS dissimilarity measure with R-F and MAST distributions, showing the number of obtained pairs of trees (y axis) with certain distance values (x axis) in 1000 trials.

This distribution leads to similar observations and conclusions. The E(1,1), E(1,2), NFC(1,1), NFC(1,2), NFC(2,1) distributions are similar or identical (the figure has been omitted). E(1,1) and R-F are again similar, however the distribution does not rise asymptotically with increasing distance value Figure 21. Both E(3,1) (Figure 22) and FS (Figure 23) look better than MAST and F-S as they take more values E(3,1) - 24, FS - 27 versus R-F - 8 and MAST - 4, which make them more discriminative.

### Unrooted leaf-labelled trees on a free leafset

**Figure 24:**
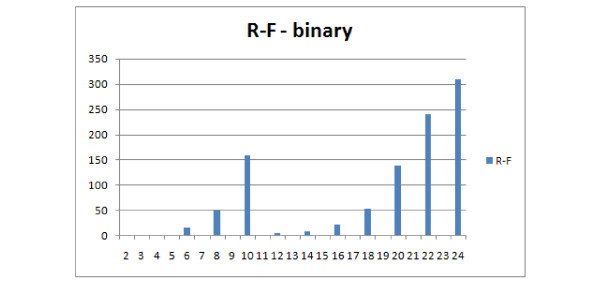
**Comparison of distributions of selected measures**. R-F distributions for binary trees on free leafset, showing the number of obtained pairs of trees (y axis) with certain distance values (x axis) in 1000 trials.

**Figure 25:**
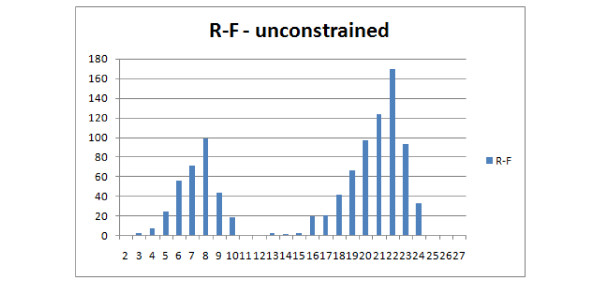
**Comparison of distributions of selected measures**. R-F distributions for unconstrained trees on free leafset, showing the number of obtained pairs of trees (y axis) with certain distance values (x axis) in 1000 trials.

**Figure 26:**
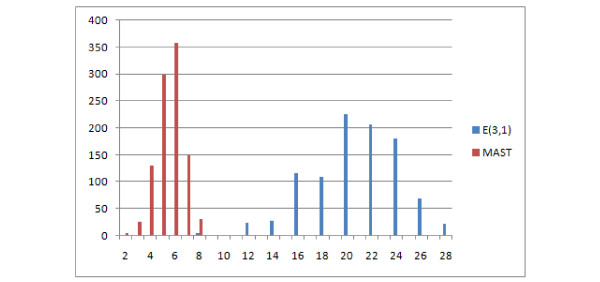
**Comparison of distributions of selected measures**. Comparison of E(3,1) distance and MAST distributions, showing the number of obtained pairs of trees (y axis) with certain distance values (x axis) in 1000 trials.

In this experiment trees with at most 8 leaves were generated. Both binary and unconstrained versions will be discussed together as the differences are only with the R-F distance. Characteristics of R-F distribution in this experiment does not recall typical R-F distribution. The main reason is that it is unsuitable for comparing trees with different leafsets as it will always return the maximum value, which will also be dependent on the number of leaves of the trees. Therefore the distribution reflects the conditional probability of selecting two trees with the same leafset(left part of graph) and trees with different leafsets (right part of graph) of Figure 24 (binary) and Figure 25 (unconstrained). As for the other distances, both E(3,1) and FS behave similarly, having a wide range of values, while E(3,1) is more regular (see Figure 26). Both E(3,1) (Figure 22) and FS (Figure 23) look better than MAST and F-S as they take more values E(3,1) - 24, FS - 27 versus R-F - 8 and MAST - 4, which make them more discriminative.

**Conclusion **: To summarise the key points of this experiment:

• The R-F distance is not very discriminative for binary trees, it is also weak for distant trees. It is not suitable for trees with different leafsets.

• The MAST distance is good for the same and different leafset, and is good both for distant and similar trees, however it is only weakly discriminative.

• The Edit distance, with the variant where cost of contraction = 1 and pruning = 3, looks very promising as it has a wide range of values and is equally good for distant and similar trees.

• The FS dissimilarity measure is similar to the Edit distance, but it does not have a very regular distribution.

• NFC here behaves like E(1,1) i.e. it is equivalent to R-F for the same leafset and equivalent to MAST for different leafsets, which is good. However it is still only very weakly discriminative.

### Dynamics of Distances

For this test, one tree is randomly generated and then the second tree is obtained with k mutation operations. Here, we observe the dynamics of distance changes with respect to number and type of mutations. Due to the nature of most of the examined distances i.e. Edit Distance, No Forced Contraction Similarity Measure, MAST and R-F, we use the following types of mutation:

• Contraction - we randomly remove a selected split

• Pruning - we randomly remove a selected leaf

• Nearest Non-Brother Interchange (NNBI).

**Figure 27:**
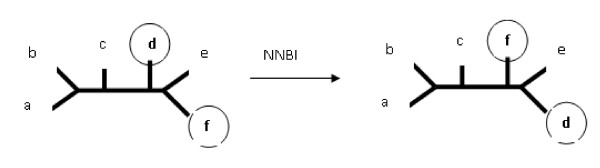
**Nearest Non-Brother Interchange of leaves f and d**.

Nearest Non-Brother Interchange (NNBI) is a modification of the NNI operation [15]. We choose the nearest leaves that are not brothers and interchange the leaves as shown on Figure 27. The motivation for such a type of mutation is that we wanted to achieve such a modification of a tree that both pruning and contraction can be used to level the changes made by the operation. Direct use of both C and P in the mutation process leads to a situation where the number of leaves changes and therefore the R-F distance is hard to be compared to. The NNBI operation can be levelled with either one contraction or one pruning operation if the cousins are 3 edges away from each other. The resulting input trees are of the same leafset so the R-F distance can also be taken in the experiments which is exactly what we wanted to achieve. For the trees shown in Figure 27, it is possible to either prune leaf f (or d) from both trees or contract splits *ef*|*abcd *and *ed*|*abcf *to make the trees identical.

**Figure 28:**
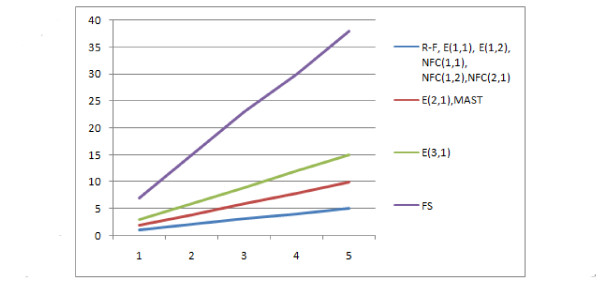
**Comparison of distances with respect to number of contraction mutations**.

To analyse the results, let us see the distances counted with respect to the contraction operation (Figure 28). All distances have similar linear dynamics and might have been simply scaled to behave identically on these data. It can be seen that all distances that have a cost of contraction equal to 1, are identical. NFC(2,1) was not identical but very similar, so it is illustrated with the same line. Those distances with a cost of c = 1 and NFC(2,1) scale the most naturally as the distance is simply equal to the number of mutations, the distance is directly proportional to the number of mutations with k = 1. Increasing the cost of contraction makes the edit distances increase quickly.

**Figure 29:**
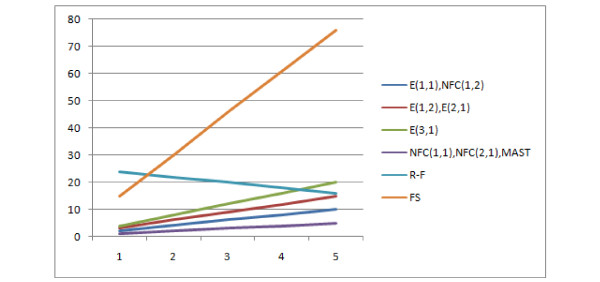
**Comparison of distances with respect to pruning mutation**.

**Figure 30:**
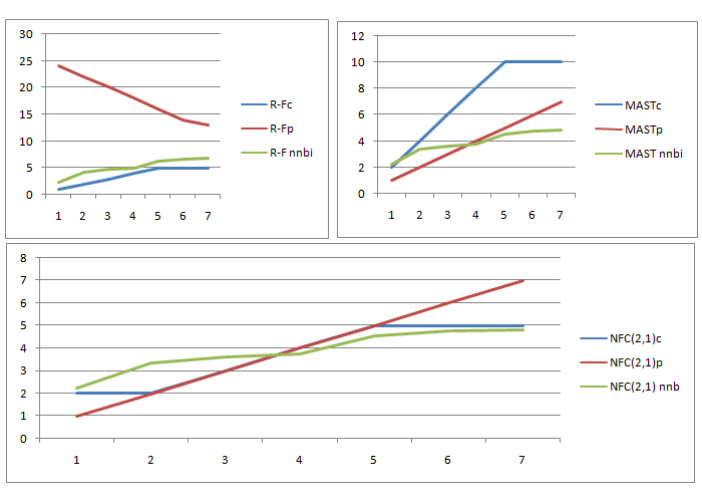
**Comparison of distances with respect to all mutations**. Comparison of distances with respect to pruning, contraction and NNBI mutation.

For a pruning mutation, the situation looks very similar (see Figure 29) i.e. all distances would have identical values if scaled, however a few things should be pointed out. The R-F distance here gets smaller with increasing number of p operations. This is because it does not work for trees with different leafsets, in such a case it returns the maximum possible value, which is lower for a smaller leafset, and this is exactly what is illustrated in the Figure. Another point is that while the R-F was the distance best scaled for contractions, MAST is the distance best scaled for pruning. This is natural because R-F uses contraction while MAST uses pruning. So if we consider not just pruning nor just contraction, but wish to use both, then the distances do not have the same dynamics. What is seen here is that NFC(2,1) scales best for both contraction and pruning. This can be visualised better when we see the reaction of distances on contraction and pruning on the same chart. In Figure 30, we can see the reaction of R-F, MAST and NFC(2,1) with respect to contraction, pruning, and Nearest Non-Brother Interchange (i.e an operation that is neither contraction nor pruning but the distance can be realised by both of these operations). We can see that only the NFC dynamics are similar irrespective of the type of mutation used.

## Conclusions

In this paper we have proposed new technique for measuring distance between leaf labelled trees on free leafset, and provided its evaluations with respect to frequent subsplit based method and other measures. The tree edit distance was proven to be a metric and has the advantage of using different costs for contraction and pruning, therefore their properties can be tuned depending on the needs of the user. It is difficult to pick the best distance measure as they all have different interesting properties and may be used in different applications. Two of the presented methods carry the most interesting properties. E(3,1) is very discriminative (having a wide range of values) and has a very regular distance distribution which is similar to a normal distribution in its shape and is good both for similar and non-similar trees. NFC(2,1) on the other hand is proportional or nearly proportional to the number of mutation operations used, irrespective of their type. All of these distances have a great advantage in that they can take different costs of contraction and pruning, therefore their properties can be tuned depending on the needs of the user. Future works will be dedicated to discovering more efficient algorithm for tree edit distance and deep experimental evaluation of tree edit consensus method for leaf-labelled trees on the same leafset.

## Authors' contributions

JK conceived the study, KW coordinated and supervised the study. All authors read and approved the final manuscript.
